# Challenges in Diagnosing and Managing the Spectrum of Primary Aldosteronism

**DOI:** 10.1210/jendso/bvae109

**Published:** 2024-06-04

**Authors:** Jun Yang, Josephine McCarthy, Sonali S Shah, Elisabeth Ng, Jimmy Shen, Renata Libianto, Peter J Fuller

**Affiliations:** Centre for Endocrinology and Metabolism, Hudson Institute of Medical Research, Clayton, 3168, Victoria, Australia; Department of Medicine, Monash University, Clayton, 3168, Victoria, Australia; Department of Endocrinology, Monash Health, Clayton, 3168, Victoria, Australia; Centre for Endocrinology and Metabolism, Hudson Institute of Medical Research, Clayton, 3168, Victoria, Australia; Department of Medicine, Monash University, Clayton, 3168, Victoria, Australia; Department of Endocrinology, Monash Health, Clayton, 3168, Victoria, Australia; Department of Endocrinology, Eastern Health, Box Hill Hospital, Box Hill, 3128, Victoria, Australia; Centre for Endocrinology and Metabolism, Hudson Institute of Medical Research, Clayton, 3168, Victoria, Australia; Department of Medicine, Monash University, Clayton, 3168, Victoria, Australia; Department of Endocrinology, Monash Health, Clayton, 3168, Victoria, Australia; Centre for Endocrinology and Metabolism, Hudson Institute of Medical Research, Clayton, 3168, Victoria, Australia; Department of Medicine, Monash University, Clayton, 3168, Victoria, Australia; Department of Endocrinology, Monash Health, Clayton, 3168, Victoria, Australia; Centre for Endocrinology and Metabolism, Hudson Institute of Medical Research, Clayton, 3168, Victoria, Australia; Department of Endocrinology, Monash Health, Clayton, 3168, Victoria, Australia; Centre for Endocrinology and Metabolism, Hudson Institute of Medical Research, Clayton, 3168, Victoria, Australia; Department of Medicine, Monash University, Clayton, 3168, Victoria, Australia; Department of Endocrinology, Monash Health, Clayton, 3168, Victoria, Australia; Centre for Endocrinology and Metabolism, Hudson Institute of Medical Research, Clayton, 3168, Victoria, Australia; Department of Endocrinology, Monash Health, Clayton, 3168, Victoria, Australia

**Keywords:** primary aldosteronism, aldosterone, renin, aldosterone-to-renin ratio, blood pressure

## Abstract

Primary aldosteronism, characterized by the dysregulated production of aldosterone from 1 or both adrenal glands, is the most common endocrine cause of hypertension. It confers a high risk of cardiovascular, renal, and metabolic complications that can be ameliorated with targeted medical therapy or surgery. Diagnosis can be achieved with a positive screening test (elevated aldosterone to renin ratio) followed by confirmatory testing (saline, captopril, fludrocortisone, or oral salt challenges) and subtyping (adrenal imaging and adrenal vein sampling). However, the diagnostic pathway may be complicated by interfering medications, intraindividual variations, and concurrent autonomous cortisol secretion. Furthermore, once diagnosed, careful follow-up is needed to ensure that treatment targets are reached and adverse effects, or even recurrence, are promptly addressed. These challenges will be illustrated in a series of case studies drawn from our endocrine hypertension clinic. We will offer guidance on strategies to facilitate an accurate and timely diagnosis of primary aldosteronism together with a discussion of treatment targets which should be achieved for optimal patient outcomes.

Primary aldosteronism (PA) is the most common endocrine cause of secondary hypertension, affecting at least 1 in 10 people living with high blood pressure (BP), and up to 1 in 3 people with resistant hypertension [1]. It increases both BP and the risk of cardiovascular, renal, and metabolic diseases [2, 3]. PA results from dysregulated adrenal production of aldosterone and therefore has targeted therapies: it can be effectively treated with mineralocorticoid receptor antagonists (MRAs) or the source of excess aldosterone can be removed with adrenal surgery [4]. Untreated PA is a large cost to society because of an avoidable disease burden and associated health care expenses, but for the individual may include a reduced quality of life for years, if not decades, before the diagnosis is made [5, 6]. An accurate diagnosis and prompt treatment of PA significantly improves BP, reduces polypharmacy, prevents target organ damage, ameliorates cardiovascular risk, and improves quality of life [7,8‐9].

A diagnosis usually begins with a screening blood test for aldosterone and renin (either renin concentration or renin activity). Increased aldosterone concentration together with feedback suppression of renin production leads to an elevated aldosterone-to-renin ratio (ARR), which then prompts aldosterone suppression testing with intravenous saline, fludrocortisone, captopril, or oral salt loading to confirm the diagnosis of PA [10]. Once confirmed, people may undergo adrenal computed tomography (CT) and adrenal vein sampling (AVS) to determine the subtype that guides subsequent management. Lateralizing forms of PA, mostly caused by aldosterone-producing adrenal adenomas, may be treated with adrenalectomy, whereas the more common bilateral forms of PA, caused by multifocal aldosterone-producing adrenal adenomas, micronodules, or hyperplasia, require lifelong medical treatment.

Diagnosis of PA can be straightforward in people who present with florid hyperaldosteronism characterized by severe hypertension and the biochemical findings of hypokalemia and markedly elevated ARR. However, in others who have less severe disease or who take commonly prescribed medications that can affect aldosterone or renin concentrations, the diagnostic process may be more complex. The diagnosis can be further complicated by intraindividual variability in aldosterone and renin as well as concurrent autonomous cortisol secretion. MRA are the mainstay of targeted therapies for PA but efficacy is dependent on the normalization of renin, whereas dosing may be limited by adverse effects. These challenges will be highlighted in a series of case studies drawn from our clinical practice that emphasize the importance of a careful diagnosis and astute monitoring of outcomes following the treatment of PA.

## Case Study 1: Investigating PA in a Patient on Multiple Antihypertensive Medications

A 48-year-old male with a 2-year history of hypertension was referred by his primary care physician with hypokalemia and a left-sided adrenal adenoma. He did not have other comorbidities. His average home-recorded BP was 147/93 mm Hg with no significant improvement on perindopril 10 mg and amlodipine 10 mg daily. His ARR and general biochemistry, apart from a potassium concentration of 3.2 mmol/L, were unremarkable, including a normal overnight 1-mg dexamethasone suppression test (Table 1). A noncontrast adrenal CT showed a left adrenal gland nodule measuring 12 mm × 8 mm with Hounsfield units of −3, in keeping with a lipid-rich adenoma.

**Table 1.** PA screening test results on and off antihypertensive medications (case 1)

Investigation, unit (reference range)	Baseline on interfering medication*^a^*	Baseline off interfering medications	Postadrenalectomy
Plasma aldosterone concentration, pmol/L (100-950)	**968**	**940**	439
Direct renin concentration, mU/L (10-50)	**85**	13	25
Aldosterone-to-renin ratio, pmol/mU (<70)	11	**72**	18
Serum potassium, mmol/L (3.5-5.2)	**3.2**	3.5	4.5

The boldface refers to values which fall outside of the reference range.

Abbreviation: PA, primary aldosteronism.

^*a*^Interfering medications at time of testing included an angiotensin-converting enzyme inhibitor (perindopril) and a dihydropyridine calcium channel blocker (amlodipine).

To optimize PA testing conditions, potassium supplementation was commenced, whereas perindopril and amlodipine were replaced with verapamil and prazosin [11]. Repeat testing 4 weeks later revealed an abnormal ARR (Table 1). A seated saline suppression test confirmed the diagnosis of PA, with an aldosterone concentration of 987 pmol/L (36 ng/dL) presaline and 739 pmol/L (27 ng/dL) postsaline. AVS lateralized to the left, consistent with a left-sided aldosterone-producing adrenal adenoma.

The patient was commenced on spironolactone while awaiting a left adrenalectomy. Verapamil and prazosin were ceased due to normalization of BP. Spironolactone was up-titrated to achieve an unsuppressed renin before laparoscopic left adrenalectomy was performed. Histopathology confirmed a 13-mm adrenocortical nodule that demonstrated diffusely strong staining for CYP11B2 (aldosterone synthase). Six weeks later, he was normotensive without any antihypertensive medications, and his potassium and ARR had returned to normal (Table 1).

## Case 1 Discussion: Impact of Antihypertensive Medications on PA Testing

Medications commonly used to treat hypertension and other cardiovascular conditions can affect the ARR, with MRA having the most profound effect on decreasing the ARR [12, 13] (Table 2). Apart from antihypertensive medications, another common confounder is the oral contraceptive pill, which can decrease renin concentration and produce a false-positive ARR [15, 16]. The Endocrine Society recommends that, where feasible and safe, washout of interfering medications should be undertaken [10]. An international survey of 33 centers from 3 continents reported that 76.7% of centers switched antihypertensive medications in preparation for PA testing [17]. Agents that markedly affect the ARR (eg, MRA, diuretic) should be withdrawn for at least 4 weeks before testing, whereas agents that affect the ARR to a lesser degree (eg, angiotensin-converting enzyme/inhibitors, angiotensin II receptor blockers, dihydropyridine calcium channel blockers) should be withdrawn for at least 2 weeks before testing [18]. Antihypertensive agents that do not significantly impact the ARR (eg, alpha receptor blockers, nondihydropyridine calcium channel blockers, moxonidine, hydralazine) are preferred during the investigation period. Medication changes require close medical supervision but may be achieved by self-titration if the patient has a reliable home BP monitor and can follow written instructions (https://www.endocrinesociety.org.au/medication-switching.asp).

**Table 2.** Effect of medications on aldosterone, renin, and aldosterone-to-renin ratio

Medication	Effect on aldosterone level	Effect on renin concentration	Effect on ARR
ACEI (eg, perindopril)	↓	↑	↓
ARB (eg, irbesartan)	↓	↑	↓
Β-blockers (eg, metoprolol)	↓	↓	↑
Central agonists (eg, clonidine, methyldopa)	↓	↓	↑
Calcium channel blockers—Dihydropyridines	↓↔	↑	↓
NSAID	↓	↓	↑
Potassium wasting diuretic	↑↔	↑	↓
Potassium sparing diuretic (including MRA)	↑	↑	↓
SGLT2i*^a^*	↔	↑	↓
Oral contraceptive pill	↔	↓	↑

Abbreviations: ↓, reduced; ↑, increased; ↔, no change; ACEI, angiotensin-converting enzyme inhibitor; ARB, angiotensin receptor blocker; ARR, aldosterone-to-renin ratio; NSAID, nonsteroidal anti-inflammatory drug; MRA, mineralocorticoid receptor antagonist; SGLT2i, sodium-glucose transport protein 2 inhibitor.

^*a*^SGLT2 inhibitor proposed mechanism: initial increase in osmotic diuresis and natriuresis results in reduction in renal perfusion pressure and sodium load at the distal tubule that can stimulate renin release [14].

There are some patients in whom interfering agents cannot be withdrawn; for example, patients with florid hyperaldosteronism may require MRA treatment to achieve a safe BP or those with labile hypertension in whom medication switching is deemed unsafe [19]. In some centers, testing is routinely performed on existing medications for pragmatic reasons, such as patient convenience or resource restraints. It is suggested that testing can be pursued if renin remains suppressed despite use of interfering medications. For patients whose initial ARR is normal on interfering medications but has a high clinical index of suspicion, remeasuring the ARR on different days may overcome intraindividual variability [20] and demonstrate an abnormal result on subsequent testing. In a retrospective cohort study of 50 patients (25 with PA, 25 with essential hypertension), half required interfering medications be continued because of severe hypertension or comorbidities [21]. Hence, a pragmatic framework for interpreting PA-related test results in the presence of interfering medications can be useful, as described in the following section.

A relatively small prospective study of 27 patients with PA and 151 patients with essential hypertension found no significant difference in the area under the receiver operated curve of the ARR on or off interfering medications when using a cutoff of 91 pmol/mU [22]. However, a larger cohort study of 331 patients (125 with PA and 206 with essential hypertension) who had ARR measured on and off interfering medications demonstrated an overall decrease in renin concentration and increase in ARR following cessation of interfering medications [23]. While on interfering medications (except β-blockers), a lower ARR cutoff of 27 pmol/mU showed comparable sensitivity and specificity to an ARR threshold of 54 pmol/mU off interfering medications. A recent retrospective cohort study by Liu et al suggested that an ARR of <19 pmol/mU on interfering medications rules out the diagnosis of PA (sensitivity 96.3% [ie, only 3.7% with PA would be missed]; specificity 61.2%) [24]. The finding of a suppressed or low renin in the setting of a medication that should cause increased renin, such as diuretics or angiotensin-converting enzyme inhibitors, can be considered suspicious for underlying PA, whereas a completely normal renin and ARR in someone who is taking medications that should cause a false-positive result (eg, β-blockers) would be more reassuring.

Although there are challenges in medication switching, this case demonstrates the importance of optimizing test conditions to accurately identify patients with PA, in this case leading to a surgical remission. When interfering medications cannot be safely withdrawn, one should still examine renin in the context of the specific agents in use or consider a lower ARR threshold.

## Case Study 2: Managing a Patient With an Inconsistent Aldosterone-to-renin Ratio

A 45-year-old male was referred by his primary care physician to an endocrinologist for investigation of secondary causes of hypertension. His father was diagnosed with hypertension at age 40 years and had a heart attack at age 44 years. The patient's home BP ranged from 129/90 mm Hg to 178/112 mm Hg. He was not taking any medications other than verapamil sustained-release 240 mg daily, nor was he taking over-the-counter drugs or other supplements. A detailed clinical assessment did not elucidate any clinical symptoms or signs of glucocorticoid or catecholamine excess. His mean seated clinic BP was 156/94 mm Hg; the initial investigations are shown in Table 3.

**Table 3.** Results of investigations for endocrine causes of hypertension (case 2)

Investigation, unit (reference range)	Results		
	Initial	Repeat (1)	Repeat (2)
Plasma aldosterone concentration, pmol/L (100-950)	350	482	294
Direct renin concentration, mU/L (10-50)	5.6	6.4	2.8
Aldosterone-to-renin ratio, pmol/mU (<70)	63	75	105
Serum potassium, mmol/L (3.5-5.2)	3.9	4.2	4.3
Estimated glomerular filtration rate, mL/min/m^2^ (>90)	>90	>90	>90
Corrected calcium, mmol/L (2.10-2.60)	2.37	—	—
TSH, mU/L (0.4-4.8)	2.4	—	—
Plasma metanephrine, pmol/L (<500)	265	—	—
Plasma normetanephrine, pmol/L (<900)	433	—	—
Plasma 3-methoxytyramine, pmol/L (<110)	100	—	—

Given his low renin concentration, he was advised to have a repeat ARR with instructions to have blood drawn in a seated position approximately 2 hours after waking. This time, his ARR was above the threshold of 70 pmol/mU, which is considered a positive screening result for PA. A third screening test was again abnormal (Table 3). During a seated saline suppression test, his plasma aldosterone concentration, measured by immunoassay, decreased from 414 pmol/L (15 ng/dL) to 190 pmol/L (7 ng/dL), with a corresponding decrease in direct renin concentration from 6.0 mU/L to 4.1 mU/L. A postsaline aldosterone above the threshold of 170 pmol/L (6 ng/dL) confirmed PA. A normal 1-mg overnight dexamethasone suppression test excluded autonomous cortisol secretion. An adrenal CT scan was normal and adrenal vein sampling was consistent with bilateral adrenal disease. Treatment with spironolactone monotherapy at a dose of 25 mg daily achieved a BP < 130/80 mm Hg and a normal renin concentration.

## Case 2 Discussion: Variability in Aldosterone, Renin and the Aldosterone-to-renin Ratio Across the Spectrum of Primary Aldosteronism

The ARR is the recommended screening test for PA [10]. An ARR >70 pmol/mU (>2.5 ng/dL:mU/L) is considered positive by our own center but the threshold varies across centers, ranging from >20 to >50 ng/dL:ng/mL/h, or >30 to >136 pmol/mU [17], depending on local experience and the assays used [25, 26]. Some centers use an additional criterion of a minimum aldosterone concentration (ranging from >140 pmol/L to >550 pmol/L) [17] to overcome the potential for a very low renin to drive up the ARR even with a low aldosterone concentration. The higher the ARR threshold or the minimum aldosterone threshold, the more likely a positive screening result will correctly identify PA (high specificity) but it will also be more likely to miss people with PA (low sensitivity) [25]. Furthermore, many physiological factors can affect ARR measurements such as salt intake, posture, time of day, menstrual cycle phase, and potassium levels [4]. As such, significant intraindividual variability in ARR has been reported with a coefficient of variation of up to 45% [27]. A recent retrospective study found that 38% (62/162) of patients confirmed to have PA on saline suppression testing had at least 1 ARR < 70 pmol/mU during the screening process [20]. Hence, a repeat ARR measurement is useful if the initial result is normal (based on local laboratory thresholds) despite clinical suspicion for PA. Although not strictly necessary, in case 2, a third ARR was measured before confirmatory testing given the discordance between the first 2 results.

It may be argued that the patient in case 2 does not have PA given the relatively low aldosterone concentration following saline infusion. Although the seated saline suppression test is highly sensitive for PA, and therefore less PA diagnoses will be missed more than the supine saline suppression test (sensitivity 88.6% vs 40.0%), there is expected to be more false-positive results (specificity 94.1% vs 100%) [28]. Nevertheless, like the ARR, there are different thresholds for post-saline aldosterone concentrations that confirm the diagnosis of PA, ranging from 140 pmol/L (5 ng/dL) to 360 pmol/L (13 ng/dL) [29, 30]. Recent research has also shown that aldosterone measured by immunoassay is often higher than that measured by liquid chromatography with mass spectrometry, resulting in the potential to cause false-positive PA diagnoses unless the cutoffs/thresholds are adjusted accordingly [31]. However, this binary approach to diagnosing PA may not capture the full spectrum of renin-independent aldosteronism [32, 33]. Baudrand et al found that in 210 normotensive people with plasma renin activity <1 ng/mL/h, 13.8% met the biochemical diagnostic criteria for PA based on 24-hour urinary aldosterone excretion despite having a normal ARR [34]. The continuum of inappropriately elevated urinary aldosterone excretion relative to salt intake was also demonstrated in 1015 hypertension trial participants in the United States [35]. Importantly, having suppressed or low renin in the context of inappropriately normal aldosterone is associated with poorer measures of cardiovascular health [36-38–39], whereas MRA is effective in reducing BP and left ventricular hypertrophy in these milder PA phenotypes [40, 41]. It is hypothesized that CYP11B2-expressing cell clusters, or adrenal micronodules, play a role in these cases that may be in the early stages of renin-angiotensin-aldosterone system dysregulation [42]. With aging and aldosterone synthesis-driver mutations, aldosterone-producing cell clusters may evolve to a more severe biochemical and clinical phenotype of PA [32, 43].

This case highlights the variability in aldosterone and renin measurements, resulting in a variable ARR. PA case detection can be optimized by conducting the blood tests under standardized conditions and on more than 1 occasion where feasible. The management of patients with mild PA, which may be considered low renin hypertension by some centers, is likely to involve the use of MRA, but more robust evidence from randomized clinical trials is needed to determine the best first-line treatment.

## Case Study 3: Approach to the Patient With Aldosterone and Cortisol Cosecretion

A 56-year-old female was referred for resistant hypertension, progressive since age 30 years, with previous episodes of hypokalemia. While on amlodipine 10 mg daily, valsartan 160 mg daily, hydrochlorothiazide 12.5 mg daily, and moxonidine 400 mcg at night, her BP was 175/90 mm Hg. She was a social smoker but did not drink alcohol. Over the preceding 12 months, she had experienced an 18-kg weight gain, skin thinning, easy bruising, and peripheral edema. On examination, she had a body mass index of 38 kg/m^2^ (weight 101 kg).

Blood tests showed hypokalemia (potassium 3.4 mmol/L) with an elevated plasma aldosterone concentration of 1050 pmol/L (38 ng/dL) and direct renin concentration of 11 mU/L. A saline infusion test failed to suppress her aldosterone concentration with an aldosterone of 455 pmol/L (16 ng/dL) and renin of 2.6 mU/L presaline, and an aldosterone of 421 pmol/L (15 ng/dL) and renin of 1.8 mU/L postsaline. Her baseline morning cortisol was 375 nmol/L (reference range 133-537) with a suppressed ACTH < 1 pmol/L. A 1-mg overnight dexamethasone suppression test was abnormal with an 8 Am cortisol of 286 nmol/L (normal <50). Her 24-hour urinary free cortisol was elevated at 328 nmol/day (normal <100), as was her midnight salivary cortisol of 11 nmol/L (normal <8). Plasma metanephrines were within the reference range. A noncontrast CT scan showed a lipid-rich right adrenal adenoma (8 Hounsfield units) measuring 32 × 30 × 23 mm and a left adrenal adenoma (13 Hounsfield units) measuring 20 × 15 × 12 mm.

AVS was performed with samples collected before and after ACTH stimulation. Using aldosterone and cortisol measurements to calculate the lateralization index, there was a small degree of right-sided aldosterone lateralization before ACTH stimulation that was abolished after ACTH administration. Using the metanephrine concentration as the denominator, there was robust right-sided lateralization of both aldosterone and cortisol production, before and after ACTH (Table 4). She underwent a right-sided adrenalectomy. Histopathology showed a benign adenomatous nodule with positive CYP11B2 immunoperoxidase staining.

**Table 4.** Adrenal vein sampling results with use of plasma cortisol or metanephrine concentrations to determine cannulation success and lateralization

Site	Aldo-sterone, pmol/L (A)	Cortisol, nmol/L (C)	Meta-nephrin, pmol/L (M)	SI based on C	SI based on M	A/C	A/M	C/M	LI based on A/C	LI based on A/M	LI based on C/M
**Pre-ACTH**											
Right adrenal vein	17 930	4046	11 880	20.1	118.8	4.43	1.51	0.34	2.95	98.9	33.5
Left adrenal vein	846	563	55 440	2.8	554.4	1.50	0.02	0.01	—	—	—
IVC	490	201	<100*^a^*	—	—	2.44	NA	NA	—	—	—
**Post-ACTH**											
Right adrenal vein	186 340	55 643	17 010	96.9	170.1	3.35	10.95	3.27	1.56	25.5	16.4
Left adrenal vein	26 620	12 411	62 065	21.6	620.7	2.14	0.43	0.20	—	—	—
IVC	1100	574	<100*^a^*	—	—	1.92	NA	NA	—	—	—

Abbreviations: A, aldosterone (pmol/L); A/C, aldosterone-to-cortisol ratio; A/M, aldosterone-to-metanephrine ratio; C, cortisol (nmol/L); C/M, cortisol-to-metanephrine ratio; M, metanephrine (pmol/L); IVC, inferior vena cava; LI, lateralization index.

^*a*^Value of 100 used to calculate the SI.

Postoperatively, she was discharged on hydrocortisone replacement and 2 antihypertensive agents that were gradually weaned and ceased by 10 months after adrenalectomy. At 12 months after adrenalectomy, her BP was 110/85 mm Hg without antihypertensive therapy. She had lost 30 kg since the surgery and all symptoms had resolved. Blood tests showed a morning cortisol of 318 nmol/L, ACTH 9.6 pmol/L, aldosterone concentration of 275 pmol/L (10 ng/dL), renin concentration of 19 mU/L, and potassium concentration of 4.4 mmol/L. This confirmed resolution of both her aldosterone and cortisol excess.

## Case 3 Discussion: Subtyping in the Setting of Aldosterone and Cortisol Cosecretion

AVS is considered the gold standard method for determining the subtype and laterality of primary aldosteronism [10]. There are variations in the methodology of AVS and interpretation of results across centers [44]. One consistent element of AVS is the use of serum cortisol concentration to determine the success of adrenal vein cannulation and to normalize aldosterone concentration in samples taken from the adrenal vein (ie, correct for differences in the dilution of adrenal blood). The selectivity index (SI), used to determine the success of cannulation, is calculated by dividing the cortisol concentration in the adrenal vein by its concentration in the peripheral vein. The lateralization index (LI) is calculated by dividing the aldosterone-to-cortisol ratio in the dominant adrenal vein by that in the contralateral adrenal vein.

Cosecretion of aldosterone and cortisol can confound AVS results because of suppressed cortisol production from the contralateral gland, leading to an inaccurate calculation of the SI and LI. Use of plasma metanephrine to determine cannulation success has been reported to have a higher rate of success than cortisol because of a higher concentration gradient between the adrenal veins and inferior vena cava [45,46‐47]. One study reported plasma metanephrine to be superior to cortisol for assessing cannulation success in the absence of ACTH stimulation [47]. In our case, the SI for the left adrenal vein was 2.8 pre-ACTH and would have incorrectly been considered failed cannulation if applying the SI threshold of 3. Using plasma metanephrine as an alternative analyte to calculate the aldosterone-to-metanephrine ratio has been shown to reduce biases associated with asymmetrical cortisol secretion for both stimulated and unstimulated AVS [48]. For unstimulated AVS, using the aldosterone-to-metanephrine ratio to calculate the LI identified cases of lateralized PA that were missed when using the aldosterone-to-cortisol ratio [48]. For our patient, the aldosterone-to-cortisol LI indicated no lateralization, whereas the aldosterone-to-metanephrine LI clearly indicated right-sided lateralization, both pre- and post-ACTH (Table 4). The interpretation of right-sided lateralization is reinforced by the strikingly higher concentration of both cortisol and aldosterone in the right adrenal vein compared to the left.

Cosecretion of cortisol with aldosterone can present on a spectrum, from mild autonomous cortisol excess that has been reported in 10% to 27% of cases of PA [49-51–52], to overt symptomatic cortisol excess, with case 3 being representative of the latter. Overt ACTH-independent cortisol excess in the presence of a unilateral adrenal adenoma would warrant surgical management, independent of aldosterone overproduction. In case 3, AVS identified the source of aldosterone and cortisol excess facilitating surgical management in the setting of bilateral adrenal adenomas.

This case demonstrates the importance of recognizing autonomous cortisol excess before AVS in patients with PA. Apart from planning for transient cortisol deficiency after unilateral adrenalectomy, awareness of cosecretion may prompt measurement of plasma metanephrine levels during AVS to maximize the accuracy of the SI and LI and minimize the risk of missing lateralized aldosterone or cortisol excess. Collection of plasma metanephrine requires separate collection tubes and transport conditions; thus, communication in advance with the AVS team about protocol changes is necessary.

## Case Study 4: Challenges in the Medical Management of PA

A 77-year-old male presented with a history of atrial fibrillation on anticoagulation and >10-year history of stage 2 hypertension. His biochemistry showed persistently elevated ARR with an aldosterone concentration ≥ 500 pmol/L (≥18 ng/dL) and suppressed direct renin concentrations ≤2 mU/L, with stage 3 chronic kidney disease and an estimated glomerular filtration rate (eGFR) of 50 mL/min/m^2^. There was no evidence of hypokalemia, autonomous cortisol excess, or an adrenal mass on CT. His medications included a cardiac selective β-blocker and a dihydropyridine calcium channel blocker. A clinical diagnosis of PA was made on the basis of elevated ARR, and spironolactone was commenced after ascertaining the patient's preference for noninvasive medical management.

On spironolactone 25 mg daily, the patient’s BP significantly improved but his renin concentration remained low at 3 mU/L. Spironolactone up-titration to ≥50 mg daily was limited by painful gynecomastia with worsening renal function (eGFR ≤ 35 mL/min/m^2^) and mild hyperkalemia (5.5 mmol/L). Spironolactone was switched to eplerenone, which was then up-titrated gradually to 50 mg twice daily to achieve adequate BP control and normalization of renin (>10 mU/L). His renal function stabilized with an eGFR of 40 mL/min/m^2^ after 6 months. His breast symptoms resolved over several months; however, he developed headaches with eplerenone, which remitted on temporary cessation but recurred on a rechallenge. Hence, eplerenone was replaced by amiloride 5 mg daily, which achieved an average home BP < 140/90 mm Hg but caused intolerable polyuria leading to drug cessation. The patient was recommenced on spironolactone with more gradual up-titration. He did not report recurrence of his gynecomastia nor mastodynia on spironolactone 37.5 mg daily, achieving an average home BP < 135/85 mm Hg, a satisfactory renin concentration of 12 mU/L, a stable average eGFR of 40 mL/min/m^2^, and normokalaemia (≤5.2 mmol/L).

## Case 4 Discussion: Key Aspects in Monitoring the Medical Treatment of PA and New Options on the Horizon

The case illustrates an elderly gentleman with a high clinical and biochemical probability of PA without hypokalemia or an adrenal mass. Furthermore, with the patient's desire for noninvasive medical management, a confirmatory test and AVS were deemed unnecessary.

MRA is the recommended medical therapy for people with nonlateralizing PA or those who do not desire surgical treatment. The treatment goals include normalization of BP, serum potassium, and renin concentration (>8-12 mU/L) or plasma renin activity (>1 ng/mL/h), as unsuppressed renin has been associated with reduced cardiovascular complications [53, 54]. In Australia and most other countries, spironolactone is commonly used as first-line therapy. An initial decline in renal function, as reflected by creatinine-based eGFR, can be observed following MRA or surgical treatment of PA because of correction of aldosterone-mediated glomerular hyperfiltration, which unmasks underlying renal dysfunction [55]. Reassuringly, the drop in eGFR does not reflect intrinsic damage to the renal tubules as biomarkers of renal tubular health are restored following targeted treatment [55] and a larger acute fall in eGFR in this setting has been associated with less decline in the longer term [56].

The “on-target” antagonism of the renal mineralocorticoid receptor (MR) can lead to hyperkalemia and further fall in renal function; thus, serum potassium and eGFR should be monitored, especially in the elderly and those with chronic kidney disease, as in this case [57, 58]. In such challenging cases, gradual and cautious up-titration of MRA dose based on responses in BP, serum potassium, and renal function are recommended while recognizing that this may limit the ability to achieve the desired target renin concentration. In our practice, we advise patients to reduce or temporarily cease MRA and monitor home BP closely during episodes of volume depletion or dehydration to prevent acute deterioration in renal function.

The “off-target” effects of blocking the androgen receptor and activating the progesterone receptor are not uncommon with spironolactone and are dose-dependent. Antiandrogenic side effects of spironolactone are common in men, especially in those with underlying androgen deficiency, and may present with worsening sexual dysfunction, mastodynia, and/or gynecomastia [59]. Therapeutic strategies include the reduction of spironolactone dose and/or the use of alternative MRA such as eplerenone, or an epithelial sodium channel blocker such as amiloride (see the following section).

In females, spironolactone is contraindicated in pregnancy because of potential feminizing effects on a male fetus; therefore, contraception is recommended in women of reproductive age who take spironolactone [60]. In premenopausal women, menstrual irregularities may occur; thus, a more selective MRA such as eplerenone may be useful. Drospirenone, a spironolactone-derived progestin, may be an alternative in this cohort; at 4 mg daily, this progestin-only oral contraceptive pill offers contraception without the potential cardiovascular side effects of ethinylestradiol [61]. It also possesses anti-MR activity at approximately 8 times the potency of spironolactone and can be combined with spironolactone to achieve the desired therapeutic effects for PA [62].

Eplerenone is an alternative MRA that is much more selective for the MR than spironolactone which helps to avoid the antiandrogenic and progestogenic side effects. However, it is weaker in potency and has a short half-time with no long-acting metabolites, thus requiring twice daily dosing. The starting dose is usually double the intended spironolactone dose and it is not uncommon for people with PA to require up to 150 mg of eplerenone a day to “unsuppress” renin and restore BP control [63, 64]. Eplerenone is unfortunately relatively expensive, which limits widespread use in many countries. Interestingly, the most common postmarketing side effect reported in Japan is headache [65], as demonstrated in our case.

Another non-MRA alternative for PA is amiloride, an inhibitor of the epithelial sodium channels in the principal cells of the collecting duct, which is the final common pathway in the action of MR activation in the distal nephron [66]. It may be used by itself or in combination with spironolactone to avoid dose-related side effects of spironolactone [9], although evidence for cardiovascular protection by amiloride in PA is lacking.

The recent development of third-generation non-steroidal MRA such as finerenone, esaxerenone, ocedurenone, and aparenone may broaden the therapeutic options for PA [67] (Table 5). Finerenone is potent and highly selective for MR with lower rates of hyperkalemia compared to the steroidal MRA (spironolactone, eplerenone). This may be attributed to the more balanced tissue distribution of the drug in the kidneys and heart, rather than the kidneys alone, as with the steroidal MRA [71]. Finerenone has been shown in 2 major randomized controlled trials to reduce progression of kidney disease and cardiovascular events in patients with type 2 diabetes and albuminuria [72, 73]. In these cohorts, the BP-lowering effects of finerenone were only modest (2-3 mm Hg in systolic BP) and the efficacy of finerenone in PA has not been studied. In contrast, newer nonsteroidal MRAs such as ocedurenone and aparenone have been shown to effectively lower systolic BP by 8 to 10 mm Hg in patients with diabetes or chronic kidney disease [74, 75]. In a PA cohort, esaxerenone, currently only available in Japan, was observed to significantly reduce systolic BP by 17.7 mm Hg [76]. The favorable pharmacology of these nonsteroidal MRAs in the absence of off-target effects holds great promise in the medical treatment of PA, and case 4 demonstrates a scenario in which they may play a role.

**Table 5.** Pharmacokinetic properties of mineralocorticoid receptor antagonists and epithelial sodium channel blocker

	Spironolactone [68]	Eplerenone [68]	Finerenone [69]	Amiloride [70]
Selectivity for MR	+	+++	++++	−
IC50/potency on MR	24 nM/+++	990 nM/+	18 nM/+++	100 nM on ENaC
Off-target effects	++	+/−	−	−
Half-life	18 h	4-6 h	2-3 h	6-9 h
BP-lowering effect	+++	++	+	+++

Abbreviations: BP, blood pressure; ENaC, epithelial sodium channel; MR, mineralocorticoid receptor;.

Finally, an emerging therapy for hypertension, aldosterone synthase inhibitors (baxdrostat and lorundrostat), have been shown to be effective for resistant hypertension in 2 phase 2 placebo-controlled trials [77, 78]. Although there is no study focusing on people with PA, by targeting aldosterone synthesis, it is likely effective in the treatment of primary and secondary aldosteronism [79].

## Case Study 5: Surgical Treatment May Not Always Lead to a Cure

A 39-year-old male was referred to an endocrine outpatient unit with resistant hypertension and hypokalemia. He was diagnosed with hypertension 5 years prior and received treatment with perindopril 10 mg daily, lercanidipine 20 mg daily, moxonidine 400 mcg daily, and prazosin 0.5 mg daily. His other comorbidities were obesity with a body mass index of 35 kg/m^2^ and obstructive sleep apnea treated with continuous positive airway pressure. He had no features of cortisol excess.

Biochemical testing showed a serum potassium of 3.2 mmol/L with normal renal function. He required supplementation with 20 tablets daily of potassium chloride 600 mg to raise his serum potassium to 3.6 mmol/L. After switching to noninterfering antihypertensive medications, his ARR was significantly elevated at 268 pmol/mU (<70), with a corresponding plasma aldosterone concentration of 1070 pmol/L (38 ng/dL) and direct renin concentration of 4 mU/L. His cortisol was <50 nmol/L following a 1-mg overnight dexamethasone suppression test.

His CT scan demonstrated a 2-cm cystic lesion in the left adrenal gland. He proceeded to AVS, which lateralized to the right adrenal gland. He had an LI of 8.6 pre-ACTH with contralateral suppression (aldosterone:cortisol ratio of the contralateral adrenal vein less than the ratio in the peripheral vein), and an LI of 2.7 post-ACTH without contralateral suppression. He proceeded to a laparoscopic retroperitoneal right adrenalectomy without postoperative complications. Histology revealed a 10-mm benign cortical adenoma.

**Figure 1.**
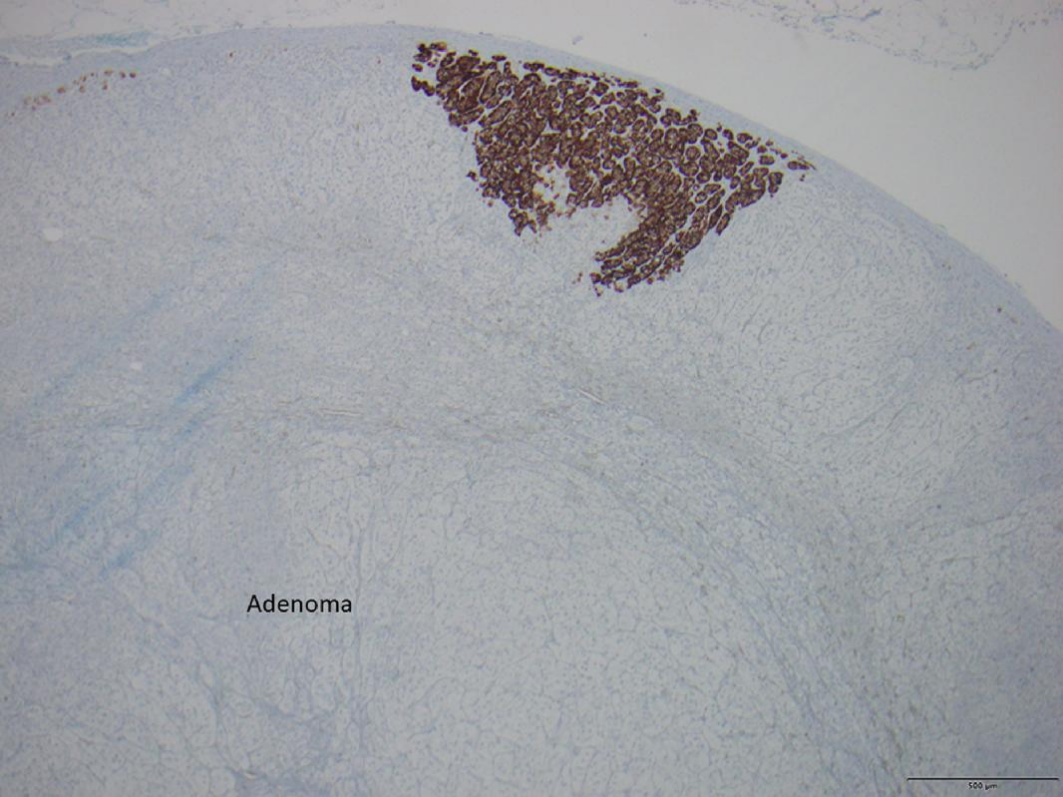
Histopathology showing an aldosterone-producing micronodule outside the adenoma, with strong positive staining for aldosterone synthase (CYP11B2, cytochrome P450 family 11, subfamily B, member 2), a crucial enzyme in aldosterone synthesis.

He was discharged on 2 antihypertensives and did not require further potassium supplementation. Over the subsequent 6 months, his BP progressively increased. Repeat testing showed a persistently elevated ARR of 155 pmol/mU with plasma aldosterone of 451 pmol/L (16 ng/dL), renin of 2.9 mU/L, and serum potassium of 4.0 mmol/L. A saline suppression test confirmed nonsuppressibility of aldosterone, and he was recommenced on spironolactone 25 mg daily. CYP11B2 staining, which subsequently became available at our facility, was requested and performed on the surgical specimen. It demonstrated absent staining in the adenoma, but positive micronodules in the adrenal cortex outside the adenoma (Fig. 1).

## Case 5 Discussion: Aspects of Follow-up for Surgically Treated PA

Clinical and biochemical outcomes following unilateral adrenalectomy for patients with PA can be assessed using the Primary Aldosteronism Surgery Outcome criteria [7] (Table 6). It is recommended that the first outcome assessment be performed 3 months postsurgery and the final outcome at 6 to 12 months postsurgery. If persistent or recurrent disease is suspected, confirmatory testing is recommended. The consensus also suggested that outcomes be reassessed annually. Our patient had partial clinical and biochemical success at 6 months, on the basis of a similar BP as before surgery with less antihypertensive medications, correction of hypokalemia, and a raised ARR with >50% decrease in the plasma aldosterone concentration. In the Primary Aldosteronism Surgery Outcome study, of 700 patients analyzed, complete clinical success was achieved in 37% of patients and complete biochemical success in 94%.

**Table 6.** PA surgical outcome consensus on outcome assessment following surgery for unilateral PA

	Clinical success	Biochemical success
Complete	Normal blood pressure without antihypertensive medication	Correction of hypokalemia (if present before surgery), *and* normalization of the aldosterone-to-renin ratio
Partial	The same blood pressure as before surgery with less antihypertensive, *or* Reduction in blood pressure with either the same or fewer antihypertensive medication	Correction of hypokalemia (if present before surgery), *and* a raised ARR with either a > 50% decrease in baseline plasma aldosterone concentration, or abnormal but improved postsurgery confirmatory test result
Absent	Unchanged or increased blood pressure with either the same amount or an increase in antihypertensive medication	Persistent hypokalemia (if present presurgery) or persistently raised ARR, with failure to suppress aldosterone secretion with a postsurgery confirmatory test

Abbreviation: ARR, aldosterone to renin ratio; PA, primary aldosteronism.

An appreciation of the underlying histopathology of PA may provide clues to differential outcomes following adrenalectomy. Unilateral aldosterone-producing adenoma and bilateral adrenal hyperplasia were traditionally described as the 2 main subtypes of PA. With the use of CYP11B2 immunostaining, a more diverse histopathologic classification, Histopathology of Primary Aldosteronism, was proposed in 2021 [80] (Table 7). Using the Histopathology of Primary Aldosteronism consensus, our patient had multiple aldosterone-producing micronodules, previously known as aldosterone-producing cell clusters, which could explain the partial clinical and biochemical response. In adrenal specimens that lacked discrete CYP11B2-positive adenoma, a higher rate of absent or partial biochemical response was reported following adrenalectomy [81]. In a recent cohort study involving patients from 7 referral centers, 16% of 283 patients who underwent AVS-guided adrenalectomy did not achieve complete biochemical remission following adrenalectomy, suggesting that asymmetrical bilateral PA could be difficult to differentiate from unilateral PA by AVS [82]. A recent study reported that contralateral suppression >50% in ACTH-stimulated AVS was a good predictor of clinical and biochemical success postsurgery [83]. In this case, contralateral suppression was only observed pre-ACTH but not post-ACTH. Variable expression of the ACTH receptor (melanocortin receptor 2) in aldosterone-producing adrenal adenomas and normal adrenocortical tissue could explain the discordant pre- and post-ACTH results. Thus, it is important to follow patients after adrenalectomy and assess their clinical and biochemical responses. In the case described, one would speculate that the left adrenal gland had similar micronodules although asymmetric aldosterone production with right-sided dominance was demonstrated at the time of AVS.

**Table 7.** Histopathology of primary aldosteronism consensus for histopathological classification of unilateral primary aldosteronism

Nomenclature	Abbreviation	Description
Aldosterone-producing adrenocortical carcinoma	APACC	Malignant neoplasm
Aldosterone-producing adenoma	APA	CYP11B2-positive solitary neoplasm ≥10 mm
Aldosterone-producing nodule	APN	CYP11B2-positive lesion <10 mm, morphologically visible with H&E staining
Aldosterone-producing micronodule (aldosterone-producing cell cluster)	APM	CYP11B2-positive lesion < 10 mm, not visible with H&E staining
Multiple aldosterone-producing nodules or micronodules (micronodular hyperplasia)	MAPN or MAPM	Multiple APN or APM
Aldosterone-producing diffuse hyperplasia	APDH	Broad and uninterrupted strip of zona glomerulosa cells with more than half staining positive for CYP11B2

Abbreviation: H&E, hematoxylin and eosin.

## Summary and Conclusion

The timely and accurate detection of PA is a rewarding process for both the patient and clinician as targeted medical or surgical treatment can effectively control BP, reduce medication burden, reduce cardiovascular risk, and improve quality of life. Often, the diagnosis and treatment are straightforward, however, as illustrated in our cases, there are caveats in making an accurate diagnosis and monitoring the efficacy of treatment.

Key take-home messages are: (1) many antihypertensive medications can significantly affect aldosterone or renin concentration and should be switched to noninterfering medications when safe and feasible to do so before PA testing; (2) multiple ARR measurements may be needed given the biological variability in aldosterone and renin production to ensure that the patient does not miss out on a potentially life-changing diagnosis; (3) concurrent aldosterone and cortisol excess should be diagnosed before AVS to plan for the measurement of alternate analytes for assessing cannulation success and lateralization; and (4) regular follow-up with the measurement of BP, electrolytes, renal function, and renin concentration is needed following treatment initiation to ensure that treatment targets are reached and sustained, or that a surgical remission, or cure, has been achieved.

Significant challenges remain in our approach to PA as the rigorous diagnostic process is not feasible for all, especially in the context of the high prevalence of renin-independent aldosteronism reported globally. There is a need for additional noninvasive strategies that accurately identify patients without surgically curable disease to fast-track medical treatment while reducing the obstacles involved in achieving improved health outcomes.

## Data Availability

Not applicable.

## Abbreviations

ARR: aldosterone to renin ratio
AVS: adrenal vein sampling
BP: blood pressure
CT: computed tomography
eGFR: estimated glomerular filtration rate
LI: lateralization index
MR: mineralocorticoid receptor
MRA: mineralocorticoid receptor antagonist
PA: primary aldosteronism
SI: selectivity index
